# What Becomes of Stillborn Children?

**Published:** 1893-08-05

**Authors:** 


### What Becomes of Stillborn Children?

At the meetings of the British Medical Association at Newcastle-on-Tyne held this week, Dr. Robert R. Rentoul, of Liverpool, read a paper on the "Proposed Registration of Stillborn Children." Want of space prevents us publishing Dr. Rentoul's paper as we should desire. Many people may be inclined to ask why the State should put itself to the expense of registering the births of stillborn children? And the question would be very pertinent if it were not for the very comprehensive significance of the word "stillborn" in this connexion. "Stillborn" stands for "murdered" children in thousands of instances every year.

How many children are disposed of annually who are supposed to have been stillborn it is impossible to say. But Dr. Rentoul obtained returns from 1,133 Burial Board cemeteries during the year 1890; and in the 1,133 cemeteries of those burial boards no fewer than 17,355 children supposed to be stillborn were interred in that one year. Of that number 4,569 were buried without any medical certificate of the cause of stillbirth. How many of those 4,569 were actually stillborn? How many had been foully murdered either during or immediately after birth?

On this, as on so many other subjects, the testimony of experts is desirable, and Dr. Rentoul has made it available. A coroner is an expert in all such matters; the coroner's "man" is probably more of an expert than even his master. Coroner Braxton Hicks is a very well known authority. His experience is that "many children who are termed stillborn are not really so, but have been born alive and died soon after, sometimes from natural causes, but also from suffocation and other illegal means. In fact, it is to be feared that many children *termed* (the italics are the coroner's own) still-born are disposed of in other ways." Now this coroner speaks with measure, as it is desirable that he should. There is no object in making out our people to be worse than they are. But we do want, if possible, to be at the real facts. If thousands of helpless infants are "disposed of," in the coroner's words, or, in plain terms, "murdered" every year, then let us know the truth, and let us deal with it.

Writers on medical jurisprudence, as well as coroners, are experts in this subject. Dr. Stevenson, one of the most eminent of these, states that "there is reason to believe that the non-registration of children born dead leads to many being disposed of as stillborn, which, in reality, came living into the world, but have died from neglect, exposure, or violence." More forcibly, Dr. Meymott Tidy, in his *Legal Medicine*, tells us that "so notorious is it that large numbers of these cases (deaths) could be averted, that some legislation is urgently needed." Finally, these opinions and statements of fact receive endorsement in the highest quarters. We are informed that "The Secretary of State has reason to believe that in some places the practice prevails of entering in the cemetery book as stillborn, children who have survived their birth by only a few hours, and over whose body no religious service has been performed." In other words, the cemetery officials in some places deliberately connive a the disposal of the bodies of children, who may have been murdered, without legal inquiry.

Now, this is in England. We are all proud of England, and accept the obligation of preserving her honour and her reputation. Let us compare her care for newly-born infants with that of certain countries which we think to be a little behind ourselves in all that is great, glorious, and good. Nearly all the great Continental countries register their stillborn children, for example, France, Austria, Prussia, Italy, and Spain. Many of the smaller countries, as Denmark, Sweden, Norway, Switzerland, Hesse, and Saxony, do the same. All the more advanced countries do it as a matter of course, and some of the least progressive, like Spain and Austria. England is conspicuous by her indifference to the newly-born, and especially to those who are born among the poor, and "those who have none to help them."

Such are some of the facts, though by no means all; but they constitute a fair sample. We feel deeply indebted to Dr. Rentoul and to men like him for unearthing them. Every self-respecting Englishman feels indebted. But what is it of a practical nature that we can do? Or rather, what is it of a practical nature that we are willing to do? What we ought to do is very obvious! We ought to hand the whole subject over to the medical profession, and to pay it adequately for satisfactory results. It is wonderful how little public use our country makes of the scientific and practical capacity of the medical profession. It is astonishing how jealous ignorant members of Parliament are of that scientific and practical capacity. Most miraculous of all is the tameness with which medical men submit to be misjudged by ignorant members of Parliament, and the meekness and timidity with which they ask to be allowed to render to the State the highest services of a cultured civilization at wages which even paupers might scorn to accept. The whole thing is a scandal to England, and a disgrace to the Parliament which regulates British affairs. Our counsel to the medical profession is to leave Parliament alone, and to instruct the public on all these painful and shameful subjects. Let the public know how helpless infants are murdered by the thousand; let self-respecting voters understand how Parliament allows all this to be done without lifting a single finger to prevent it, because its prevention would annually cost some twenty or thirty thousand pounds; let all sorts and conditions of men know that cemetery authorities, secure in the indifference of their Governmental superiors, laugh to scorn medical men who burn to wipe away this shameful disgrace from the scutcheon of our country. Englishmen as a nation are proud and honourable; Englishmen in Parliament are often both mean and ignorant, and have not the sense to be ashamed of their ignorance or their meanness. We hope Dr. Rentoul and those who are working with him will blow a trumpet throughout the land which will make every self-respecting Englishman ashamed of his country and of its brutal indifference to the helpless and the newly born. Medical inspection and medical registration of all births of every kind would put an end to this intolerable scandal in a single year.

